# Functional Characterization of the Dendritically Localized mRNA Neuronatin in Hippocampal Neurons

**DOI:** 10.1371/journal.pone.0024879

**Published:** 2011-09-14

**Authors:** Elaine L. Oyang, Bonnie C. Davidson, Winfong Lee, Michael M. Poon

**Affiliations:** Department of Biology, Harvey Mudd College, Claremont, California, Untied States of America; Centre national de la recherche scientifique, University of Bordeaux, France

## Abstract

Local translation of dendritic mRNAs plays an important role in neuronal development and synaptic plasticity. Although several hundred putative dendritic transcripts have been identified in the hippocampus, relatively few have been verified by *in situ* hybridization and thus remain uncharacterized. One such transcript encodes the protein neuronatin. Neuronatin has been shown to regulate calcium levels in non-neuronal cells such as pancreatic or embryonic stem cells, but its function in mature neurons remains unclear. Here we report that neuronatin is translated in hippocampal dendrites in response to blockade of action potentials and NMDA-receptor dependent synaptic transmission by TTX and APV. Our study also reveals that neuronatin can adjust dendritic calcium levels by regulating intracellular calcium storage. We propose that neuronatin may impact synaptic plasticity by modulating dendritic calcium levels during homeostatic plasticity, thereby potentially regulating neuronal excitability, receptor trafficking, and calcium dependent signaling.

## Introduction

Local translation of mRNAs in neuronal dendrites provides a means for rapidly eliciting site-specific changes in protein levels during neuronal development and synaptic plasticity. Dendritic localization of poly-A containing RNAs and translation machinery, such as ribosomes and translation factors, enables protein synthesis hundreds of microns from the soma [1], [2], [3], [4]. Gene expression profiling of isolated dendrites identified as many as 450 putative dendritic mRNAs in the hippocampus [5], [6], [7], [8]. However, only a handful of mRNAs, including *Arc* (activity regulated cytoskeletal protein), *Eef1α* (eukaryotic elongation factor 1 α), and *CaMKIIα* (Ca^2+^/CaM dependent kinase IIα) have been verified as being dendritically localized and/or translated in response to stimuli, such as those that induce long-term potentiation (LTP), long-term depression (LTD), or homeostatic plasticity [9], [10], [11], [12], [13].

One uncharacterized dendritic mRNA encodes the protein neuronatin (NNAT) which was first identified in embryonic rat brain and subsequently shown to be enriched in isolated dendrites [5], [14]. NNAT is expressed in rat as two alternatively spliced isoforms, encoding an 81 or 54 amino acid protein (NNATα or β) [14]. Its levels are highest early in brain development, with the NNATα isoform being expressed at E7–10 and the β isoform appearing at E11–14, during the onset of neurogenesis. NNATα and β levels continue to increase during neurogenesis (between E16–19) and decrease postnatally [15]. The *Nnat* gene is also maternally imprinted and contains a neuron-restrictive silencer element [16], [17]. Non-neuronal data from pancreatic beta and 3T3-L1 cells shows that NNAT resides in the endoplasmic reticulum (ER) and modulates intracellular Ca^2+^stores [18], [19]. NNAT is strikingly similar to phospholamban (PLN), an ER-resident Ca^2+^ regulator found in cardiac muscle. Both proteins bear α-helical membrane domains and highly basic cytoplasmic tails [14]. However, even though the mechanism by which PLN regulates Ca^2+^ by SERCA pump inhibition has been studied extensively, much less is known about the cellular and molecular function of NNAT in mature neurons, particularly within the dendrite [20].

Local intracellular Ca^2+^ concentrations are tightly controlled and compartmentalized in neurons [21]. Due to its potent effects, cytoplasmic Ca^2+^ is rapidly cleared by mechanisms such as extrusion into the ER or mitochondrial uptake [22], [23], [24]. Aberrant Ca^2+^ levels may result in abnormal synaptic development and learning-related plasticity, and may contribute to cognitive disorders such as Fragile X Syndrome [25], [26], [27], [28], [29]. Calcium signaling, particularly in dendrites and spines, has been tied to LTP and LTD induction, as well as spine morphology [22], [26], [30], [31], [32]. Local translation of dendritically localized transcripts such as calmodulins, CaMKIIá, visinin-like protein-1, NMDA (N-methyl-D-aspartic acid), and AMPA (2-amino-3-(5-methyl-3-oxo-1,2- oxazol-4-yl)propanoic acid) receptors may be a means for regulating dendritic Ca^2+^ signaling [5],[33],[34],[35],[36],[37]. Although uncharacterized in neurons, NNAT may also belong to this group due to the dendritic localization of its mRNA and its ability to regulate intracellular Ca^2+^ [18],[19].

Since local translation and Ca^2+^ regulation are critical during synaptic plasticity, and given its ability to modulate intracellular Ca^2+^ in non-neuronal cells, we asked how NNAT translation was locally regulated and if it might be involved in dendritic Ca^2+^ signaling in mature hippocampal neurons. We also examined a potential interaction between *Nnat* mRNA and FMRP (Fragile X Mental Retardation Protein), an RNA binding protein that regulates dendritic mRNA localization and translation, and whose absence or loss-of-function is thought to underlie Fragile X syndrome [38],[39],[40]. Here, we report that NNAT is indeed dendritically translated in mature neurons during homeostatic plasticity and that it likely regulates dendritic calcium by modulation of intracellular Ca^2+^ stores.

## Results

### Localization of neuronatin mRNA and protein in mature hippocampal dendrites

**Figure 1:**
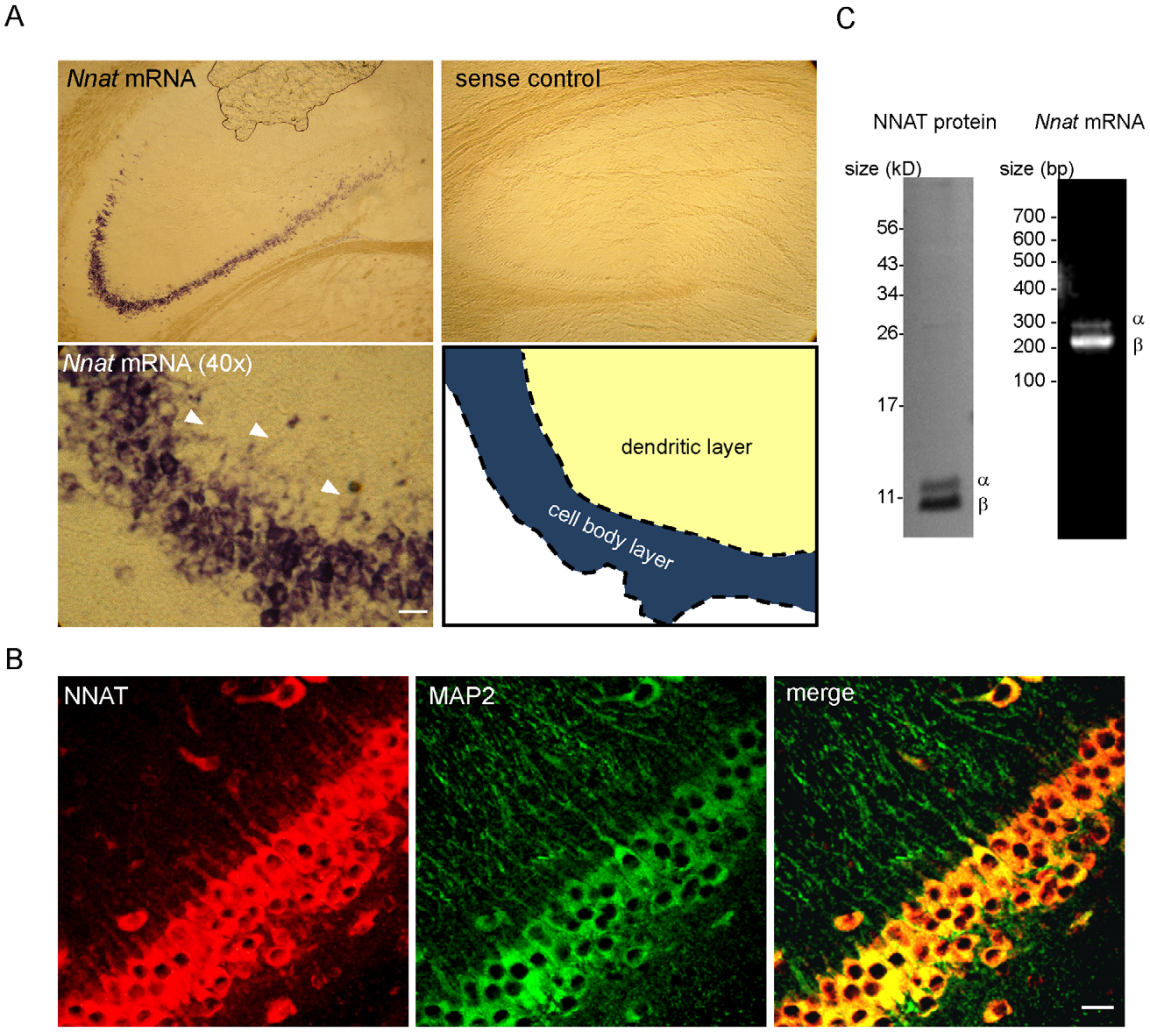
Neuronatin is expressed in the P21 rat hippocampus. (**A**) *Nnat in situ* hybridization (ISH) in hippocampus visualized using alkaline phosphatase. *Nnat* mRNA is present in hippocampal regions CA2 and 3. Arrowheads denote dendritic localization. Scale bar: 20 µm. Schematic of cell body and dendritic layer boundary in 40× image is shown (*bottom right panel*). (**B**) NNAT immunofluorescent staining in the CA2 region of hippocampus showing dendritic localization, NNAT (red), MAP2 (green). Scale bar: 20 µm. (**C**) *left*, Western blot for NNAT and *right*, RT-PCR for *Nnat* mRNA using rat hippocampal tissue showing both α and β isoforms.

To understand the function of NNAT in mature neurons, we first examined NNAT expression in rat hippocampal tissue (P21). At this age, neurons in the hippocampus have formed synaptic connections and are capable of displaying forms of learning-related and homeostatic plasticity [41],[42]. *In vivo*, *Nnat* mRNA was somatodendritically expressed, but its expression was restricted primarily to hippocampal CA2 and CA3 regions (Fig. 1A). Our data agrees with the Allen Mouse Brain Atlas (http://www.brainatlas.org), which further shows expression in the caudate, hypothalamus, and amygdala. Immunohistochemistry in rat hippocampal slice also showed somatodendritic NNAT protein expression in CA2 and CA3 as demonstrated by co-localization with the somatodendritic marker MAP2 (microtubule-associated protein 2) (Fig. 1B, Fig. S1A). NNAT intensity was strongest in proximal dendrites. NNAT antibody specificity was verified by immunostaining HeLa cells overexpressing NNAT (Fig. S1B). Both NNAT isoforms were present in the P21 and adult hippocampus as confirmed by Western blot and RT-PCR (Fig. 1C and Fig. S1C).

**Figure 2:**
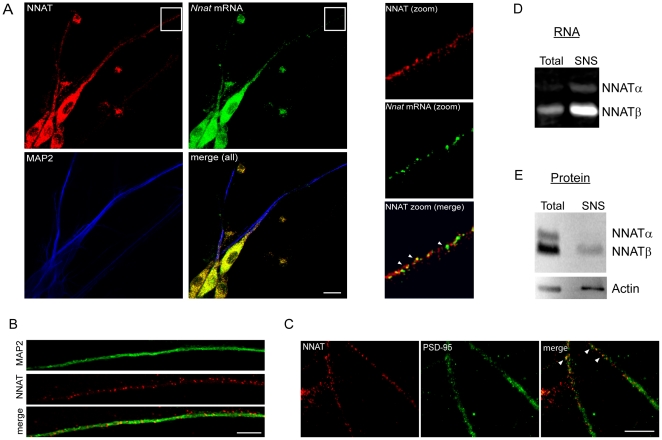
NNAT mRNA and protein are present in dendrites and synapses. (**A**) NNAT protein (red) in cultured hippocampal neurons; *Nnat* mRNA (green); somatodendritic marker, MAP2 (blue). Scale bar: 10 µm. Zoom of region boxed in white. Arrowheads denote sites of co-localization. (**B**) NNAT puncta along the dendrite as shown by immunofluorescence, MAP2 (green), NNAT (red), and merge. Scale bar: 10 µm. (**C**) *Right*, immunocytochemistry shows partial NNAT co-localization with synapses using antibodies against NNAT (red) and the synaptic marker PSD-95 (green), arrowheads in merge denote sites of co-localization. Scale bar: 10 µm. (**D**) NNATα and â mRNAs are enriched in synaptoneurosomes (SNS) as analyzed by RT-PCR (25 cycles) using equal amounts of total or SNS RNA. (**E**) NNAT protein levels in total hippocampal (total) versus SNS as assessed by immunoblot. Actin levels were used as a loading control.

We next used fluorescent *in situ* hybridization (FISH) combined with immunocytochemistry (ICC) to examine the intracellular localization of *Nnat* mRNA and protein in mature hippocampal cultures. Both were observed in distal dendrites (>150 µm) as demonstrated by co-localization of *Nnat* mRNA with MAP2 (Fig. 2A, Fig. S2A). NNAT puncta were also observed along the length of the dendrite, but only partially co-localized with the synaptic marker PSD-95, suggesting that NNAT expression is not restricted to synapses (Fig. 2B and C). NNAT did not co-localize with the axonal marker β III tubulin by immunofluorescence (data not shown). NNAT mRNA and protein were observed in both excitatory and inhibitory neurons as determined by cell morphology [43].

To examine synaptic enrichment, we assessed levels of *Nnatα* and *β* mRNA and protein in synaptoneurosomes (SNS), a biochemically enriched synaptic preparation [11],[44],[45]. Interestingly, enrichment of both *Nnatα* and *β* transcripts was not reflected at the protein level, implying that translational control may play a significant role in regulating synaptic NNAT expression (Fig. 2D). PSD-95 (a synaptic marker) and histone 3 (a somatic marker) protein levels were used to verify synaptoneurosome enrichment relative to whole tissue (Fig. S2B).

### Dendritic NNAT translation in response to TTX/APV treatment

**Figure 3:**
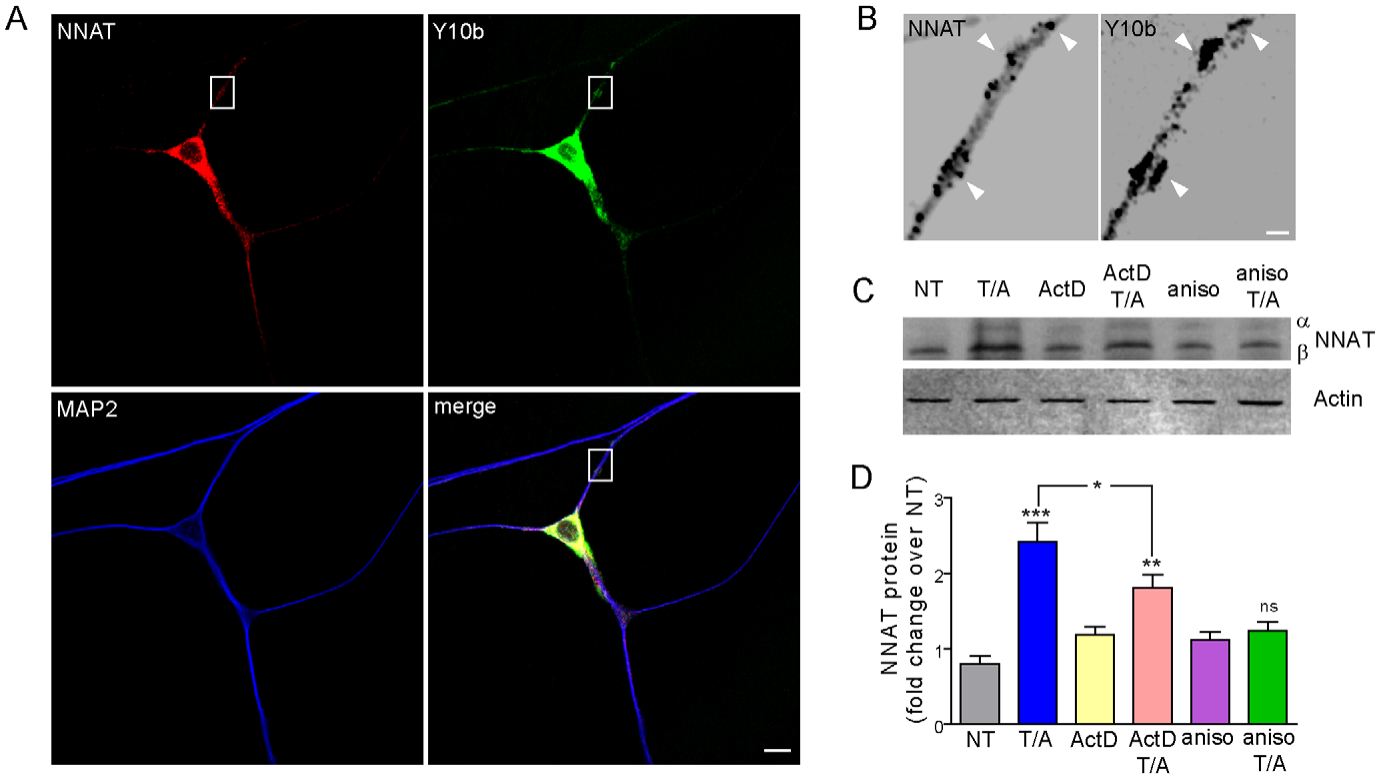
Cell-wide NNAT protein levels are induced following TTX/APV treatment. (**A**) Immunofluorescent staining in cultured hippocampal neurons using an antibody for NNAT (red), the 5.8S RNA antibody Y10b (green), and MAP2 (blue). Scale bar: 10 µm. (**B**) Magnified view of boxed region in (**A**) showing localization of NNAT and Y10b signal. Grayscale used to enhance contrast. Arrowheads denote sites of NNAT and ribosome co-localization. Scale bar: 1 µm. (**C**) NNAT is translationally regulated by TTX/APV as examined by immunoblot using lysates from hippocampal cultures treated with TTX/APV for 8 hrs in the presence or absence of transcriptional or translational inhibitors. Actin was used as loading control. (**D**) Quantification of total NNAT protein levels following treatment (expressed as fold change over no treatment (NT). Values were all n = 5 (3 independent experiments), where *** p<0.001, ** p<0.01, * p<0.05).

To determine whether *Nnat* might be locally translated, we first examined if NNAT protein co-localized with dendritic translation hot spots, characterized as discrete ribosome-containing sites along the dendrite [46]. Co-immunofluorescent staining of NNAT and ribosomes (using the Y10b antibody, which recognizes the 5.8S ribosomal subunit) showed co-localization between dendritic NNAT puncta and ribosomes in cultured hippocampal dendrites (Fig. 3A and B) [47].

NMDA receptor blockade with APV ((2*R*)-amino-5-phosphonovaleric acid) in the presence of TTX (tetrodotoxin), induces a rapid, local protein synthesis dependent form of homeostatic plasticity [41],[48]. Since TTX/APV treatment increases synaptic Ca^2+^ permeability, we wanted to examine whether NNAT, with its putative role in Ca^2+^ regulation, might also be translated in response to TTX/APV treatment [11],[41],[49],[50]. We observed a 2.4-fold increase in total NNAT levels after 8 hrs of TTX/APV in cultured hippocampal neurons (Fig. 3C and D). To determine if this induction was transcription-dependent, neurons were pretreated with a transcriptional inhibitor, actinomycin D (ActD). ActD pretreatment reduced TTX/APV-induced NNAT levels by 25%, implying a transcriptional component to the cell-wide increase in NNAT (Fig. 3C and D). We also pretreated neurons with a protein synthesis inhibitor, anisomycin, to ensure that NNAT induction was translation-dependent. As expected, anisomycin pretreatment completely abolished any increase in NNAT protein levels (Fig. 3C and D).

**Figure 4:**
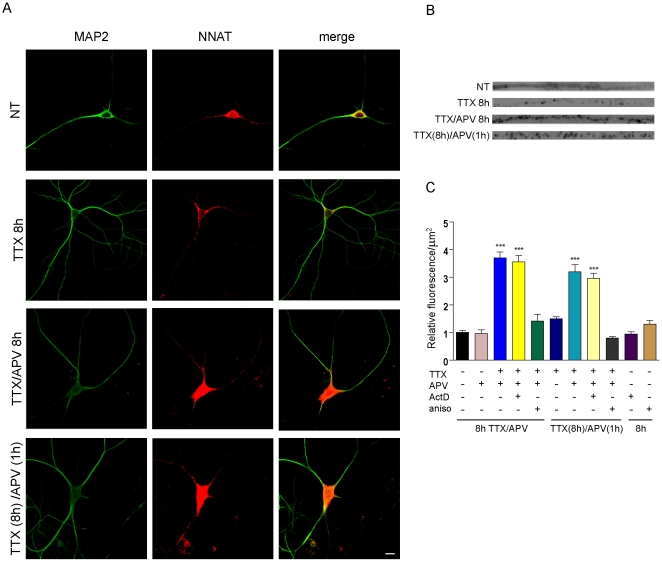
Neuronatin is dendritically translated in response to TTX/APV. (**A**) Cultured hippocampal neurons were immunostained for MAP2 and NNAT after no treatment, or treatment with 8 h TTX, 8 h TTX/APV, or TTX (8 h)+APV(1 h). Scale bar: 10 µm. (**B**) Magnified 30 µm straightened dendritic segments taken from neurons treated as in (A) showing NNAT distribution. Images were grayscaled to enhance contrast. (**C**) Quantification showing NNAT fluorescence in response to TTX/APV as a function of dendritic area (in µm^2^). All values were compared to no treatment (NT). Sample size: NT, n = 39; 8 h APV, n = 14; 8 h TTX/APV, n = 30; ActD+8 h TTX/APV, n = 30; aniso+TTX/APV, n = 12; 8 h TTX alone, n = 38; TTX (8 h)+APV (1 h), n = 18, TTX (8 h)+APV(1 h) ActD, n = 22; TTX (8 h)+APV(1 h) aniso, n = 27, actinomycin D (ActD), n = 20, anisomycin (aniso), n = 12. *** p<0.001). Each condition was performed using at least 3 independent batches of cultures.

To specifically determine whether the increase in NNAT represents dendritic protein synthesis, we performed immunofluorescent staining in cultured neurons using antibodies against NNAT and MAP2 (Fig. 4A and B). Upon TTX/APV treatment, we observed a 3.3-fold increase in dendritic signal. Importantly, even in the presence of ActD, we continued to observe a 3-fold increase in dendritic NNAT levels following TTX/APV. By contrast, pretreatment with the translation inhibitor anisomycin completely blocked this increase. Neither ActD nor anisomycin alone had any effect on NNAT baseline levels (Fig. 4C). These data indicate that an increase in translation, not transcription, is responsible for the elevated dendritic NNAT levels in response to TTX/APV treatment.

Since 8 hrs of TTX/APV treatment allows ample time for proteins to be somatically synthesized then transported to distal dendritic sites, we sought to further delineate the dendritic contribution to the NNAT increase by using short inhibition of NMDAR activity (with APV) following action potential blockade as shown by Sutton et al. [41]. We treated neurons with TTX for 8 hrs, applied APV only during the last hour, and observed an anisomycin-sensitive 2.2-fold increase in dendritic NNAT levels; TTX alone had no effect (Fig. 4A–C). This effect persisted even during transcriptional inhibition with ActD, supporting the notion that NNAT is dendritically translated (Fig. 4A and C). Similar effects were also observed using another translation inhibitor, cycloheximide (Fig. S3).

**Figure 5:**
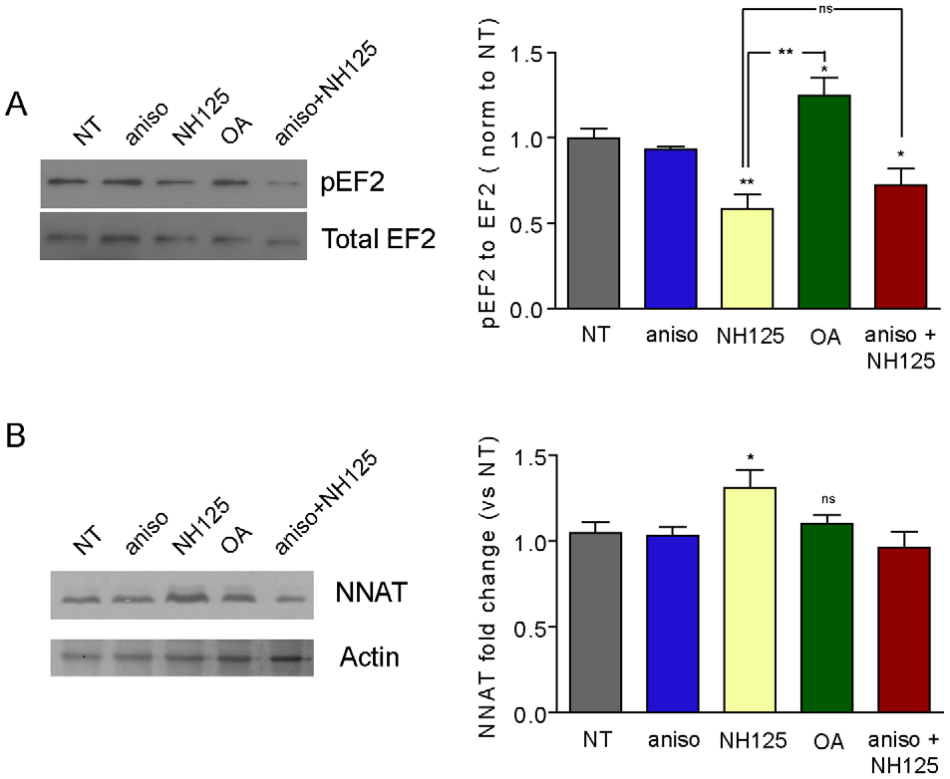
NNAT translation in synaptoneurosomes is regulated by EF2 phosphorylation. (**A**) SNS were treated with anisomycin (aniso), NH125 (an EF2 kinase inhibitor), 2 nM okadaic acid (OA) (an inhibitor of PP2A), or anisomycin+NH125, followed by immunoblot of phosphorylated (pEF2) and total EF2. Values were graphed as a ratio of phospho- to total EF2 and normalized to no treatment (n = 6 per group). (**B**) Total NNAT levels in synaptoneurosomes treated as in (A). Values were normalized to actin and compared to the no treatment group. Sample size was n = 7 (4 independent experiments) per group. ** p<0.01, * p<0.05).

During TTX/APV treatment, NMDAR-mediated Ca^2+^ influx is inhibited, suppressing EF2K (elongation factor 2 kinase) activity, resulting in enhanced translation [51]. To see if TTX/APV-induced NNAT translation could occur as a result of modulating EF2 phosphorylation, we treated SNS with either NH125 (an EF2K inhibitor) or 2 nM okadaic acid (OA), which inhibits EF2 dephosphorylation via protein phosphatase 2a [51],[52],[53]. SNS provide the advantage of being rapidly obtainable from tissue and are amenable to pharmacological manipulation and biochemical analysis [54]. We first confirmed pharmacological control of EF2 phosphorylation and observed that levels of phospho-EF2 decreased in response to NH125 and increased as a result of OA treatment (Fig. 5A). In response to NH125 treatment, we observed a 1.3-fold increase in NNAT levels; no significant increase was observed in response to okadaic acid (Fig. 5B). Inhibition of translation with anisomycin attenuated the NH125-induced increase in NNAT protein. Although stimuli other than TTX/APV blockade may also influence EF2 phosphorylation, our data show that NNAT can be translated at the synapse in an EF2-dependent manner.

### Neuronatin overexpression increases baseline calcium levels

**Figure 6:**
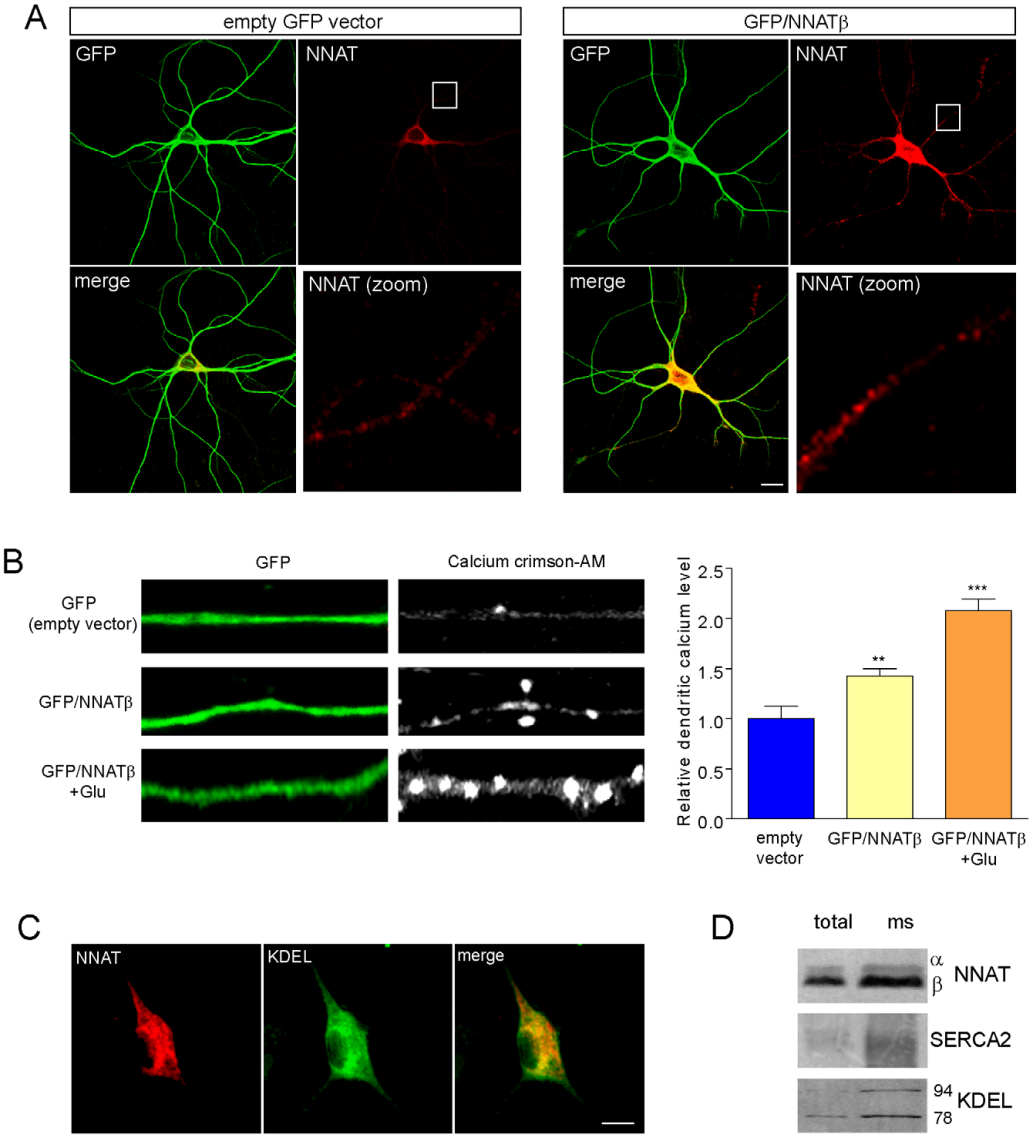
NNATβ overexpression results in elevated dendritic Ca^2+^. (**A**) Neurons were transfected with the empty GFP vector (left) or the dual expression GFP/NNATβ (right), allowed to express for 8 hrs, and immunostained for NNAT (red) and GFP (green). Scale bar: 10 µm. (**B**) *Left*, neurons transfected with empty vector or GFP/NNATβ were loaded with Calcium Crimson-AM, and dendritic GFP and Calcium Crimson signals were acquired. GFP/NNATβ transfected cells were also treated with 40 µM glutamate (5 min) following Calcium Crimson loading to further induce Ca^2+^ levels. Calcium Crimson signal was grayscaled to enhance contrast. Values expressed as ratios of Calcium Crimson normalized to GFP fluorescence (empty vector, n = 12 dendrites, 4 neurons; GFP/NNATâ, n = 12 dendrites, 6 neurons; GFP/NNATβ+Glu, n = 10 dendrites, 5 neurons, **p<0.01, ***p<0.001). Experiments were performed using 2 independent batches of cultures. (**C**) NNAT is localized to the endoplasmic reticulum (ER). Cultured neurons co-immunostained with NNAT (red) and an ER marker against KDEL (green). Scale bar: 5 µm. (**D**) NNAT is 1.8-fold enriched in microsomes (ms) generated from hippocampi compared to total protein. Microsome enrichment was assessed using antibodies against ER markers Grp94 (2.1-fold enrichment), Grp78 (2.7-fold enrichment), and SERCA2 (3.0-fold enrichment).

NNAT overexpression in non-neuronal cell types modulates intracellular Ca^2+^ storage, resulting in elevated cytoplasmic Ca^2+^ levels [18],[19].To see if NNAT could regulate dendritic Ca^2+^ levels, we overexpressed NNATβ, the predominant isoform, in mature hippocampal cultures followed by loading with Calcium Crimson-AM, a BAPTA-based Ca^2+^ indicator dye [55],[56]. NNATβ was overexpressed using a dual expression construct containing GFP and the full length rat *Nnatβ* mRNA sequence (including 5′ and 3′ UTRs to preserve translational control and localization) driven by separate promoters (GFP/NNATβ) (Fig. 6A). Neurons overexpressing NNATβ (as identified by GFP fluorescence) exhibited a 1.3-fold increase in dendritic Ca^2+^ levels compared to those transfected with the empty vector (Fig. 6B). A brief five minute glutamate application was used to verify that Ca^2+^ had not reached ceiling levels.

### NNAT regulates calcium by antagonizing SERCA pump activity

Given the structural similarities between NNAT and phospholamban, and the presence of SERCA in neuronal dendrites and synapses, we hypothesized that NNAT might function as a SERCA pump regulator (Fig. S2B) [57]. ER localization of NNAT has been previously observed in other cell types, but has not been examined in mature neurons [18],[58]. We verified NNAT localization to neuronal ER by co-immunostaining NNAT with a KDEL ER-marker antibody and observed perinuclear co-localization, consistent with ER distribution (Fig. 6C). Microsomes (an ER-enriched preparation) generated from rat hippocampi, also showed a 1.8-fold enrichment of NNAT (Fig. 6D). Microsome purity was verified by immunoblotting with the KDEL (which recognizes Grp78 and Grp94) or SERCA2 antibody (Fig. 6D).

**Figure 7:**
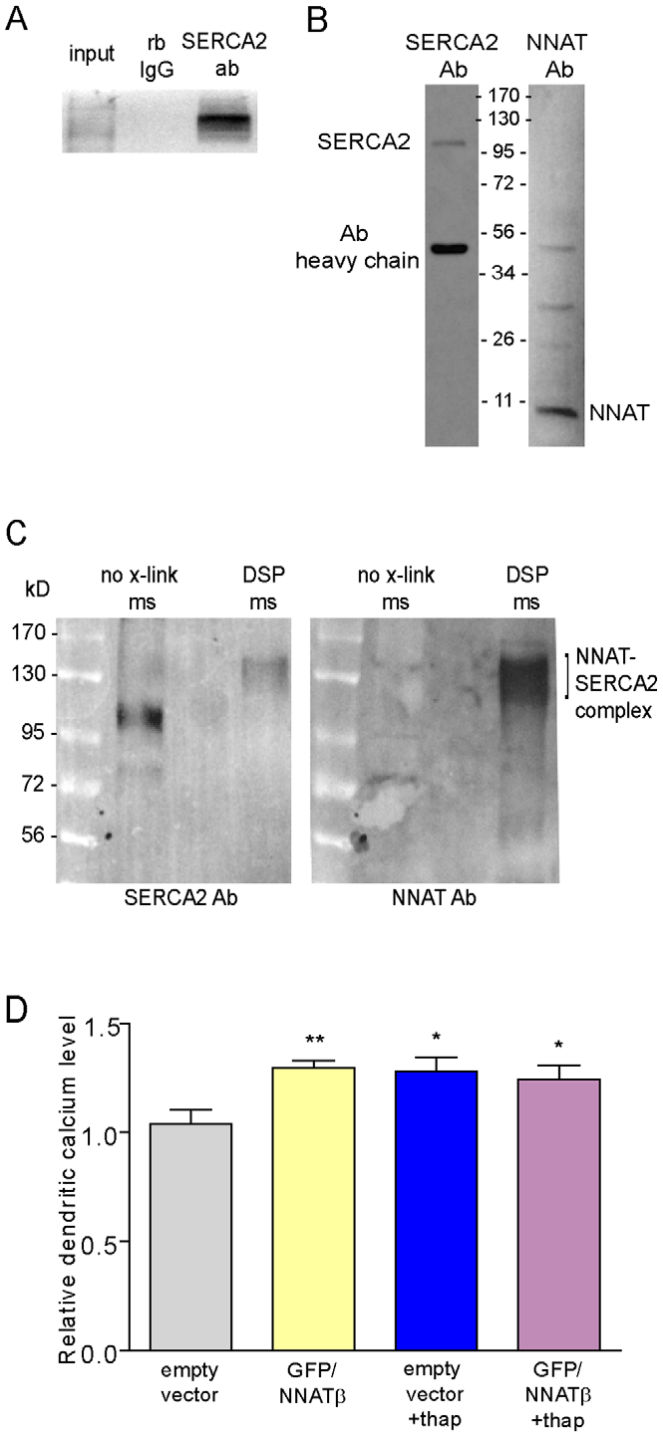
Neuronatin is associated with SERCA2 in the hippocampus. (**A**) SERCA2 antibody immunoprecipitation efficiency compared to rabbit IgG. 2.5% of input or immunoprecipitated samples were loaded. (**B**) NNAT was co-immunoprecipitated from microsomes using SERCA2 antibody. Pulldown was confirmed by immunoblotting with SERCA2 or NNAT antibody. (**C**) Microsomes were crosslinked with dithiobis [succinimidyl propionate] (DSP) followed by SERCA2 immunoprecipitation and immunoblot analysis. SERCA2 antibody (*left*) recognizes a 114 kD band in uncrosslinked microsomes and a size-shifted complex at 120–150 kD after crosslinking and IP. *Right*, membrane was stripped and reprobed with NNAT antibody, revealing the presence of NNAT within the 120–150 kD complex. (**D**) Overexpression of GFP/NNATβ occludes Ca^2+^ elevation by thapsigargin (GFP/NNATβ+thap), as measured using Calcium Crimson-AM, suggesting that NNAT antagonizes SERCA pump activity (empty vector: n = 24 dendrites, 14 neurons; GFP/NNATâ: n = 33 dendrites, 14 neurons; empty vector+thap: 18 dendrites, 8 neurons; GFP/NNATβ+thap: 19 dendrites, 8 neurons; GFP/NNATβ+thap+Glu: 19 dendrites, 9 neurons. **p<0.01, *p<0.05). Experiments were performed using at least 2 independent batches of cultures.

We next examined whether NNAT interacts with the SERCA pump. Using an antibody against SERCA2, the predominant neuronal isoform, we were able to co-immunoprecipitate (co-IP) NNAT from hippocampal microsomes suggesting a possible association (Fig. 7A and B). However, to show a more direct association, we first crosslinked microsomes using DSP (dithiobis [succinimidyl propionate]), followed by co-IP using the SERCA2 antibody. In uncrosslinked microsomes, we detected SERCA2 at 114 kD by Western blot (Fig. 7C, left). Upon crosslinking and co-IP, however, we observed a size-shifted SERCA2-containing complex at ∼120–150 kD (Fig. 7C, left). Re-probing of the same blot with the NNAT antibody revealed that this complex also contained NNAT (Fig. 7C, right).

Given this association, we hypothesized that the elevation in dendritic Ca^2+^ following NNAT overexpression was due to increased NNAT inhibition of SERCA pump activity. If this were true, we would expect NNAT overexpression to occlude Ca^2+^ induction by a SERCA inhibitor, such as thapsigargin. Indeed, no additive Ca^2+^ increase was observed following thapsigargin treatment in neurons transfected with GFP/NNATâ, while in empty GFP vector transfected neurons, thapsigargin increased dendritic Ca^2+^ by 1.23-fold (Fig. 7D). Similar to before, overexpression of GFP/NNATâ resulted in a 1.25-fold increase in dendritic Ca^2+^. To exclude the possibility that NNATβ overexpression induced a Ca^2+^ ceiling effect, following thapsigargin, we treated GFP/NNATâ transfected neurons with an additional 5 min pulse of glutamate (to activate extracellular Ca^2+^ influx or intracellular Ca^2+^ release) and observed a 1.54-fold increase in Ca^2+^ over the GFP/NNATβ transfected baseline levels (data not shown).

### Fragile X Mental Retardation Protein binds *Nnat* mRNA

**Figure 8:**
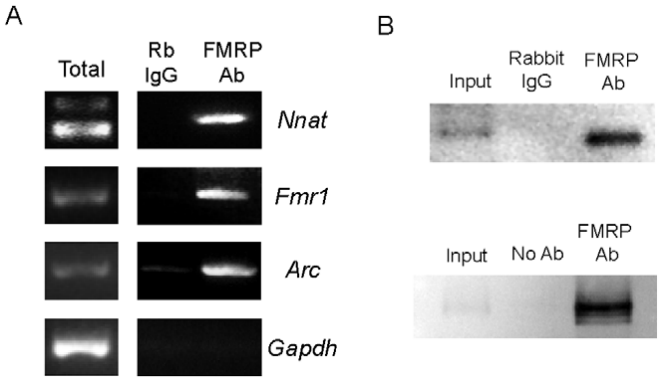
*Nnat* mRNA associates with FMRP. (**A**) RNA co-immunoprecipitation (non-UV crosslinked) followed by RT-PCR using FMRP antibody or rabbit IgG for *Nnat* mRNA. Known FMRP binding targets, *Arc* and *Fmr1*, are enriched in the FMRP immunoprecipitated sample, whereas *Gapdh*, a non-target, is not. Based on product size, *Nnatβ* was the predominant FMRP-binding isoform. RT-PCR using total hippocampal RNA is shown in the left column. (**B**) FMRP immunoprecipitation efficiency was tested using 2.5% input or immunoprecipitated hippocampal protein, probed with FMRP antibody and compared to rabbit IgG or no antibody.

A screen for mRNAs associated with FMRP (Fragile X Mental Retardation protein) by Miyashiro et al., suggested a putative association between FMRP and *Nnat* mRNA [38]. Recent work has also shown that the loss of FMRP impairs TTX/APV-induced homeostatic plasticity [59]. Using hippocampal tissue, we confirmed that *Nnat* mRNA associates with FMRP by RNA co-IP using an FMRP antibody (Fig. 8A). Binding specificity was verified by enrichment of two known FMRP binding targets, *Arc* and *Fmr1* [10],[60] and the lack of G*apdh* enrichment in the immunoprecipitated sample (Fig. 8A) [39],[61]. Immunoprecipitation of FMRP protein was specific as assessed by rabbit IgG and no antibody negative controls (Fig. 8B). However, when attempting to co-immunoprecipitate *Nnat* mRNA and FMRP from hippocampal tissue at high stringency using ultraviolet crosslinking (which is specific for protein-nucleic acid interactions) and an SDS-containing wash buffer, we were unable to detect a direct association between FMRP and *Nnat* mRNA [38],[62]. This lack of association by UV crosslinking suggests the intriguing possibility that an unknown intermediate, such as a protein or RNA species, may facilitate the interaction between FMRP and *Nnat* mRNA.

## Discussion

Although high embryonic and early postnatal expression has suggested significant roles for NNAT during neuronal development, its function in mature neurons has not been examined. Here, we show that *Nnat* mRNA is expressed and dendritically translated during homeostatic plasticity in mature hippocampal neurons. Moreover, we have demonstrated that it regulates dendritic Ca^2+^ levels by antagonizing SERCA pump activity. Given the importance of Ca^2+^ signaling during neuronal events, together with data that *Nnat* mRNA associates with FMRP, we propose that NNAT may play a crucial role in synaptic and possibly cognitive function.

The presence of ER throughout dendrites and synapses provides a readily accessible Ca^2+^ source, potentially allowing NNAT to modulate local intracellular Ca^2+^, and thus influence site-specific events, such as LTP and LTD [21],[63],[64]. Since the ER is continuous between the dendritic shaft and synapse, transport along the ER membrane could facilitate the localization of dendritically translated NNAT to the synapse. Thus, induction of synaptic NNAT levels would not be limited exclusively to translation at the synapse. Rather, such a mechanism would allow dendritic protein synthesis to contribute to synaptic NNAT levels as well [65],[66].

TTX/APV-induced homeostatic plasticity regulates dendritic translation by modulating the state of EF2 phosphorylation [51]. Our data here shows that synaptic *Nnat* mRNA translation is indeed sensitive to EF2 phosphorylation; however, NNAT induction was relatively modest, suggesting that other factors may be involved. Retinoic acid has recently been implicated in TTX/APV mediated homeostatic plasticity [11]. Molecularly, RARα (retinoic acid receptor α) binds to consensus motifs in the 5′UTR of certain mRNAs thus repressing their translation [67]. Unpublished data from our lab suggests that while RA does affect NNAT protein levels, *Nnat* mRNA does not bind RARá, and is probably not subject to direct translational regulation by RARα. We are currently investigating additional molecular mechanisms at play, including possible *cis*-acting motifs in the 5′ and 3′ untranslated regions that might underlie the translational control or localization of *Nnat* mRNA. Our data also shows that TTX/APV results in a transcription-dependent increase in NNAT. Although we have yet to investigate the underlying mechanism, previous reports have shown that TTX-induced homeostatic plasticity can induce transcription via CaMKIV [68].

Many FMRP-regulated mRNAs are present in dendrites and it has been proposed that their aberrant translation or mislocalization is an underlying cause of Fragile X syndrome [69],[70],[71]. We observed here that *Nnat* mRNA associates with FMRP, but likely through an indirect interaction. Recently, Edbauer and colleagues reported that a subset of microRNAs associate with FMRP to regulate synapse structure and function [61]. Such an intermediate might also participate in the FMRP-*Nnat* interaction. Interestingly, certain hallmarks of the *Fmr1^−/−^* mouse (a model of Fragile X syndrome), including enhanced mGluR-LTD, prolonged epileptiform bursts in hippocampal CA3, and elongated dendritic spines are consistent with the notion of perturbed Ca^2+^ signaling [26],[69],[72]. We are currently conducting studies in the *Fmr1* ^−/−^ mouse to investigate whether these might also be linked to perturbed NNAT levels.

Using dendritic protein synthesis to regulate Ca^2+^ signaling presents an attractive mechanism as precise, spatial control of Ca^2+^ is essential for synapse formation, elimination and various forms of learning-related plasticity [21]. NNAT is well-suited as one such regulator as its local synthesis would allow for sustained changes in cytoplasmic Ca^2+^ levels. However, the precise molecular events governing the interaction between NNAT and the SERCA pump remains an important question to be answered. That NNAT contains several putative sites of posttranslational modification, including phosphorylation, implicates the involvement of additional signaling pathways. Modification at such sites could facilitate the integration of multiple signaling events at or near the synapse, resulting in the tuning of intracellular Ca^2+^ signals and any associated downstream pathways [73].

In summary, our data support that NNAT functions as a dendritic Ca^2+^ regulator whose levels are locally controlled in an activity-dependent manner. Its potential involvement in cognitive disorders, such as Fragile X syndrome, makes NNAT an appealing candidate for future study. Interestingly, phenylketoneuria patients, some of whom display autism-like symptoms, also exhibit abnormally high levels of NNAT [74],[75]. We posit therefore, that understanding the action and regulation of NNAT may shed light on the molecular basis of certain forms of cognitive impairment, particularly those associated with aberrant Ca^2+^ signaling. In a broader sense, our study also underscores a relationship between local translation and Ca^2+^ signaling, demonstrating the functional richness of dendritically localized mRNAs and the pressing need for their characterization.

## Materials and Methods

### Antibodies

Information on antibodies can be found in the Supplementary Information section.

### Primers and constructs

Primer sequences and cloning strategies can be found in the Supplementary Information section.

### Animals

Sprague-Dawley rats used in these experiments were housed at the Joint Science Department and handled according to guidelines outlined and approved by the Institutional Animal Care and Use Committee at the Joint Science Department of the Claremont Colleges. Animals were euthanized using CO_2_ followed by decapitation and tissue collection.

### Calcium imaging

Calcium imaging was performed as described in Korkotian and Segal [76],[77]. Further details can be found in the Supplementary Information section.

### SERCA2 crosslinking and co-immunoprecipitation

Microsomes were prepared from P21 hippocampal tissue as described previously [78]. For immunoprecipitation, SERCA2 antibody or rabbit IgG was prepared by prebinding with Protein A/G beads (Santa Cruz Biotechnology, Santa Cruz, CA) equilibrated in NP-40 lysis buffer (1% Nonidet P-40 in PBS, pH 7.4) containing protease inhibitor. DSP (dithiobis [succinimidylpropionate])(Pierce, Rockford, IL) was dissolved in dry DMSO at 25 mM and used on microsomes at 2 mM and incubated for 2 h on ice and quenched at 4°C overnight in 20 mM Tris, pH 7.5. Crosslinked microsomes were pelleted at 140,000× g at 4°C for 1 h. The supernatant was removed and the microsomes resuspended in NP-40 lysis buffer with protease inhibitor and lysed at 4°C overnight with agitation. Lysates were precleared with Protein A/G beads, then incubated with antibody-bound beads at 4°C overnight and samples prepared in 2× SDS sample loading sample (crosslinked samples were prepared in the absence of 5% β-mercaptoethanol).

### FMRP RNA co-immunoprecipitation

RNA co-immunoprecipitation was performed as described previously [79] with the following exceptions: hippocampal tissue was Dounce homogenized in RNAse-free lysis buffer containing 0.5% Nonidet P-40 in PBS, 300 mM NaCl, 2 mM MgCl_2_, 2 mM CaCl_2_, 20 mM Tris, pH 7.4, 5 mM DTT, 1 mg/mL yeast tRNA, protease inhibitors and 40 U/mL RNAseOut (Invitrogen, Carlsbad, CA) and cleared by centrifugation. Antibody was prebound to protein A/G beads in lysis buffer. Lysates were also precleared using Protein A/G beads equilibrated in lysis buffer. The FMRP antibody used for IP (Cell Signaling, Cat#4317) was against a peptide spanning amino acids 536–593 of human origin, which is similar to that used by others [80]. After overnight incubation with lysates, antibody-bound beads were washed three times with lysis buffer, then twice with lysis buffer containing 650 mM NaCl as previously described [79]. RNA was extracted directly from beads using Trizol (Invitrogen, Carlsbad, CA). Immunoprecipitation by UV crosslinking was performed as described previously [67].

### Microscopy

All images were taken using either a Zeiss Pascal LSM 510 confocal microscope and LSM software or a Nikon Eclipse 90i epifluorescent microscope using Metamorph software (Universal Imaging, Downington, PA).

### Statistical Analysis

All statistics were performed using one-way analysis of variance followed by post hoc analysis with a Newman-Keuls multiple comparison test.

Additional experimental procedures can be found in the Methods S1.

## Supporting Information

Figure S1 (**A**) NNAT expression extends into distal dendrites in hippocampal slice. Top left, 40× merged image from Figure 2B, MAP2 (green), NNAT (red). Top right, magnified image (in order to highlight NNAT expression >100 µm from the soma (arrowhead). Asterisk denotes a possible interneuron in the dendritic layer. Scale bar: 10 µm. (**B**) NNAT antibody is specific for NNAT by immunocytochemistry. HeLa cells were transfected with NNATβ or empty vector (pCI-Neo), then immunostained using the NNAT antibody (green). Cells were counterstained with propidium iodide (red). Scale bar: 50 µm. (**C**) *left*, Western blot for NNAT and *right*, RT-PCR for *Nnat* mRNA using P21 and adult (7 month old) rat hippocampal tissue showing both α and β isoforms.
(TIF)

Figure S2 (**A**) Sense control for *Nnat* fluorescent *in situ* hybridization. *Top left panel*, *Nnat* sense control (green), *top right panel*, NNAT immunofluorescence (red), *bottom left*, MAP2 (blue), *bottom right*, merge. Scale bar: 10 µm. (**B**) Synaptoneurosome enrichment was assessed by Western blot using antibodies against Histone 3 (cell body marker) or PSD-95 (synaptic marker) on equal amounts of total or synaptoneurosome (SNS) protein samples. SERCA2 is also present in SNS.
(TIF)

Figure S3 TTX/APV-induced local NNAT synthesis is inhibited by the translation inhibitor, cycloheximide. Quantification summarizing NNAT fluorescence as a function of dendritic area (in µm^2^) in response to TTX/APV treatment in the presence or absence of cycloheximide. All values were compared to no treatment (NT) using one-way ANOVA followed by Newman-Keuls multiple comparison test (sample size: NT, n = 34 dendrites; 8 h TTX/APV, n = 39 dendrites; actinomycin D (ActD), n = 35 dendrites; ActD+8 h TTX/APV, n = 33 dendrites; cycloheximide (CHX), n = 11 dendrites; CHX+TTX/APV, n = 13 dendrites, *** p<0.001). Experiments were performed using at least 3 batches of independent cultures.
(TIF)

Methods S1 (DOCX)
