# Supplementary figures and images for: Functional Characterization of the Dendritically Localized mRNA Neuronatin in Hippocampal Neurons
Source: PLoS One. 2011 Sep 14;6(9):e24879. doi: 10.1371/journal.pone.0024879 (PMC3173491; doi:10.1371/journal.pone.0024879)

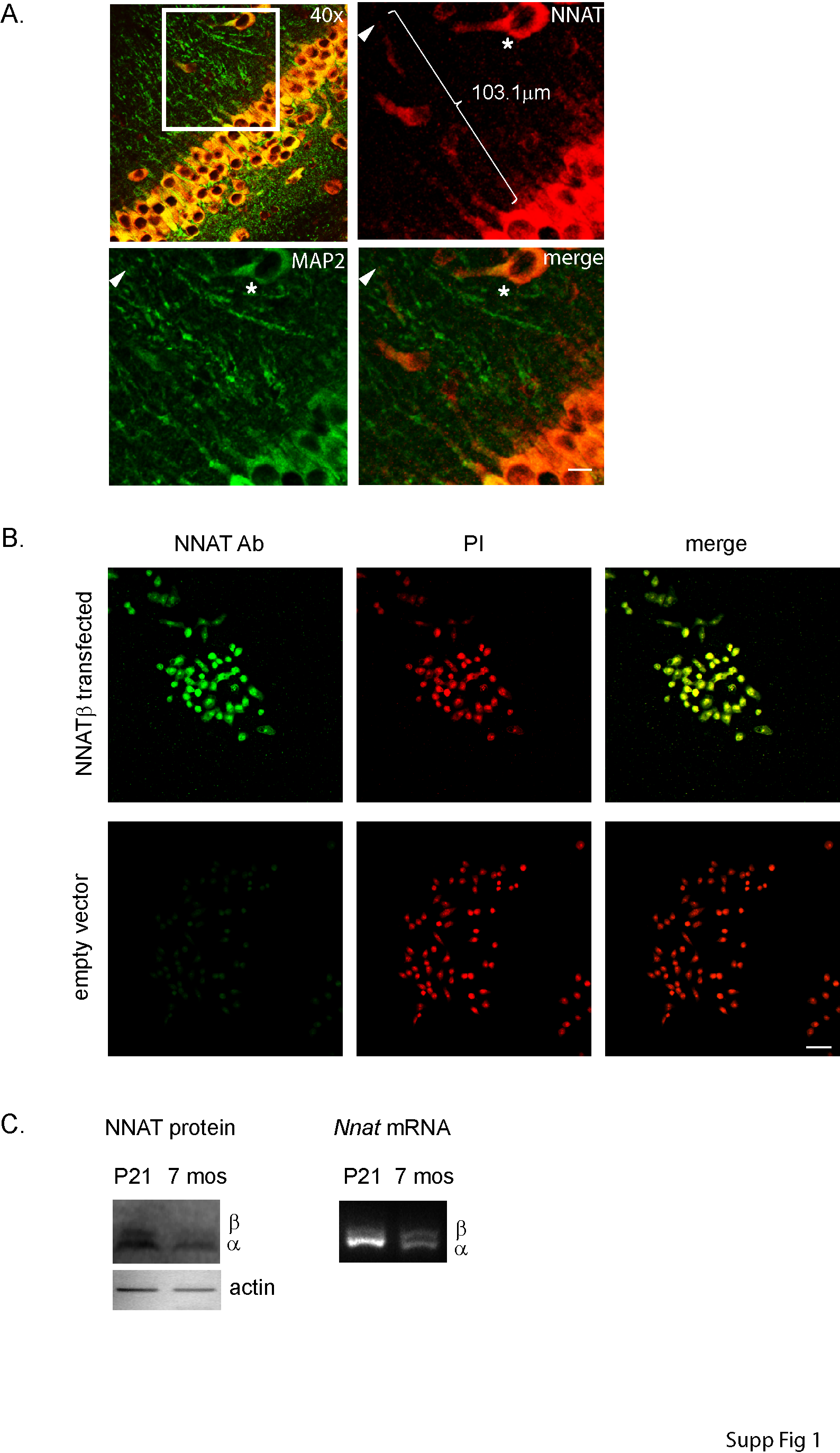

Supplement: Figure S1 — (A) NNAT expression extends into distal dendrites in hippocampal slice. Top left, 40× merged image from Figure 2B, MAP2 (green), NNAT (red). Top right, magnified image (in order to highlight NNAT expression >100 µm from the soma (arrowhead). Asterisk denotes a possible interneuron in the dendritic layer. Scale bar: 10 µm. (B) NNAT antibody is specific for NNAT by immunocytochemistry. HeLa cells were transfected with NNATβ or empty vector (pCI-Neo), then immunostained using the NNAT antibody (green). Cells were counterstained with propidium iodide (red). Scale bar: 50 µm. (C) left, Western blot for NNAT and right, RT-PCR for Nnat mRNA using P21 and adult (7 month old) rat hippocampal tissue showing both α and β isoforms. (TIF) [file pone.0024879.s001.tif]

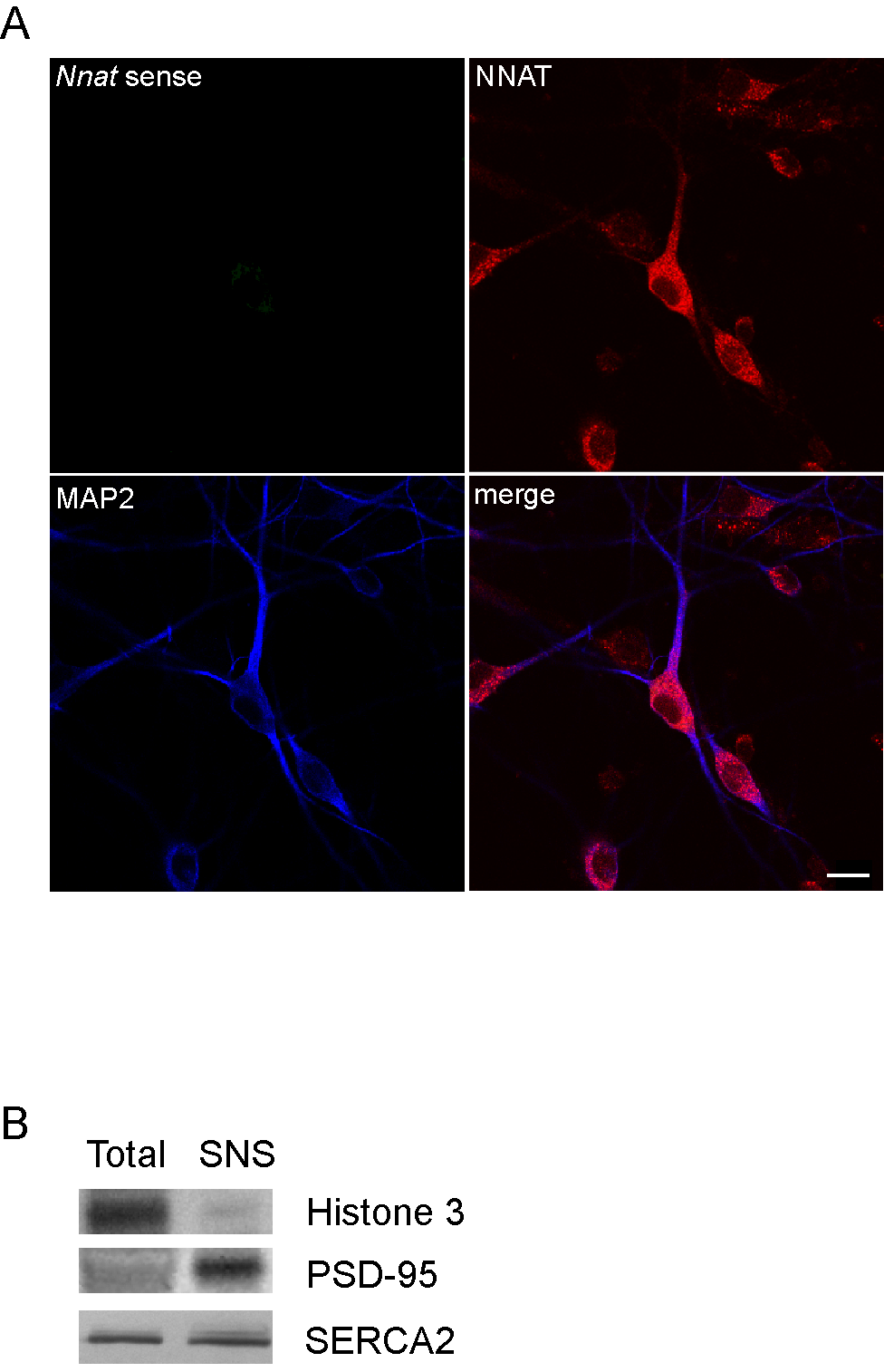

Supplement: Figure S2 — (A) Sense control for Nnat fluorescent in situ hybridization. Top left panel, Nnat sense control (green), top right panel, NNAT immunofluorescence (red), bottom left, MAP2 (blue), bottom right, merge. Scale bar: 10 µm. (B) Synaptoneurosome enrichment was assessed by Western blot using antibodies against Histone 3 (cell body marker) or PSD-95 (synaptic marker) on equal amounts of total or synaptoneurosome (SNS) protein samples. SERCA2 is also present in SNS. (TIF) [file pone.0024879.s002.tif]

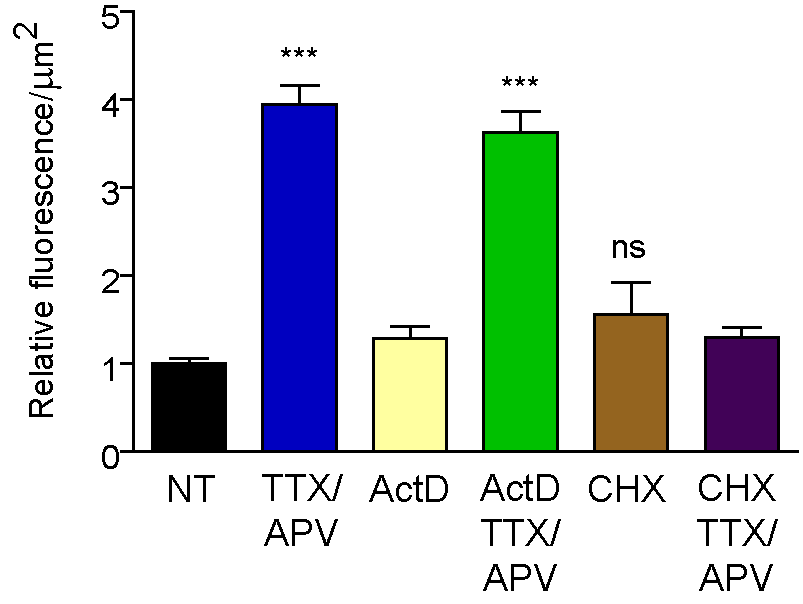

Supplement: Figure S3 — TTX/APV-induced local NNAT synthesis is inhibited by the translation inhibitor, cycloheximide. Quantification summarizing NNAT fluorescence as a function of dendritic area (in µm2) in response to TTX/APV treatment in the presence or absence of cycloheximide. All values were compared to no treatment (NT) using one-way ANOVA followed by Newman-Keuls multiple comparison test (sample size: NT, n = 34 dendrites; 8 h TTX/APV, n = 39 dendrites; actinomycin D (ActD), n = 35 dendrites; ActD+8 h TTX/APV, n = 33 dendrites; cycloheximide (CHX), n = 11 dendrites; CHX+TTX/APV, n = 13 dendrites, *** p<0.001). Experiments were performed using at least 3 batches of independent cultures. (TIF) [file pone.0024879.s003.tif]
