# Supplementary material for: Functional Characterization of the Dendritically Localized mRNA Neuronatin in Hippocampal Neurons
Source: PLoS One. 2011 Sep 14;6(9):e24879. doi: 10.1371/journal.pone.0024879 (PMC3173491; doi:10.1371/journal.pone.0024879)
Supplement: Methods S1 — (DOCX) [file pone.0024879.s004.docx]

**Supplementary Information**

***Antibodies***

Fluorescent conjugated secondary antibodies were purchased from Jackson Immunoresearch (West Grove, PA) and used at 1:1000 for immunocytochemistry, and 1:250 for immunohistochemistry. HRP-conjugated secondary antibodies were purchased from Jackson Immunoresearch (West Grove, PA) and used at 1:3000. Rabbit IgG was purchased from Jackson Immunoresearch (West Grove, PA)

Primary antibodies and applications were as follows:

| **Primary antibody** | **Vendor** | **Working dilution (application)** |
| --- | --- | --- |
| MAP2, mouse monoclonal | Sigma, St Louis, MO | 1:1000 (ICC)  1:250 (IHC)  1:500 (Western) |
| NNAT, rabbit polyclonal | Abcam, Cambridge, MA | 1:1000 (ICC)  1:250 (IHC)  1:500 (Western) |
| Actin, mouse monoclonal | Chemicon, Temecula, CA | 1:5000 (Western) |
| ATP2a2/SERCA2, rabbit polyclonal | Cell Signaling, Beverly, MA | 1:1000 (Western)  1:50 (IP) |
| KDEL marker, rabbit polyclonal | Santa Cruz Biotechnologies, Santa Cruz, CA | 1:2000 (ICC)  1:500 (Western) |
| MAP2, rabbit polyclonal | Chemicon, Temecula, CA | 1:1000 (ICC) |
| FMRP, rabbit polyclonal | Cell Signaling, Beverly, MA | 1:500 (Western)  1:50 (IP) |
| GFP, rabbit polyclonal | Abcam, Cambridge, MA | 1:500 (ICC) |
| Pan histone H3 CT, mouse monoclonal | Millipore, Billerica, MA | 1:10,000 (Western) |
| PSD95, mouse monoclonal | Affinity Bioreagents, Golden, CO | 1:1000 (Western)  1:500 (ICC) |

ICC - immunocytochemistry; IHC - immunohistochemistry; IP – immunoprecipitation.

***Primers and constructs***

All primers are listed in the 5’ to 3’ direction.

To clone full length NNAT mRNA from rat cDNA we used the forward primer: TATAGAATTCGCGAACCCTTGCTCTCGA, and the reverse primer: TATAGTCGACTGGTGCACCCCCACTGCC.

For semi-quantitative PCR:

| NNAT Fwd  NNAT Rev | ATGGCCGCAGTGGCAGCA  CGTTGGGGGCTCGGTGCCT |
| --- | --- |
| Fmr1 Fwd  Fmr1 Rev | ATGGATCCCTGCAGAGCACCTCC  TCACCTCATGCCCTGTGCCATCT |
| GAPDH Fwd  GAPDH Rev | CCCTTCATTGACCTCAACTA  CCAAAGTTGTCATGGATGAC |
| Arc (rat) Fwd  Arc (rat) Rev | GAGAGCTGAAAGGGTTGCAC  CTGGCACCCAAGACTGGTAT |

Riboprobes for *Nnat* FISH were generated using PCR primers described in Poon et al., 2006. Riboprobes for *CaMKIIα* were generated by PCR amplifying using primers, Fwd: CAGCCACTGTATCCAGCAGA, Rev: GGGTTGATGGTCAGCATCTT.

Plasmids used for riboprobe generation were TA cloned into pGEM-T Easy (Promega, Madison, WI) or TOPO TA (Invitrogen, Carlsbad, CA) and transcribed using SP6, T7 or T3 RNA polymerase depending on insert direction and vector. GFP/NNATβ was cloned from the full length rat sequence (Genbank ID:32140177) and inserted into the plasmid pFAN (gift from Lu Chen). pFAN consists of pCI-Neo (Promega, Madison, WI) containing a PGK driven-EGFP inserted using BamHI and EcoRV. NNATβ was inserted into the pCI-Neo multiple cloning site using NheI and SalI. For HeLa cell transfections, NNATβ was cloned into pCI-Neo using the same restriction sites.

***Hippocampal cell culture***

Dissociated rat hippocampal cultures were prepared from Sprague-Dawley rats, postnatal day 0, as described in Goslin and Banker, 1999 with modifications [1]: Neurons were plated and grown for 5 days in media containing Neurobasal-A (Invitrogen, Carlsbad, CA), B-27 serum supplement (Invitrogen), 5% fetal bovine serum (FBS, Hyclone, Logan, UT), 25µM glutamate (Sigma, St. Louis, MO), 25µM β-mercaptoethanol (Fisher, Fairlawn, NJ) and Glutamax (Invitrogen, Carlsbad, CA). After 5 days in culture, 1/3 of the media was replaced with Neurobasal-A containing 4µM cytosine arabinoside (Sigma, St. Louis, MO), Glutamax, B-27 and 5% FBS. Cultures were used between 12 and 14*div.* TTX (2µM) and APV (40µM) were purchased from Ascent Scientific (Princeton, NJ). Anisomycin (40µM) and Actinomcyin D (8µM) were purchased from Sigma (St Louis, MO).

***Fluorescent* in situ *hybridization (FISH)***

FISH in cultured neurons was performed as described in Poon et al., 2006 or in slice according to Guzowski and Worley, 2001, with the following exceptions: Hybridization buffer consisted of 50% formamide (Fisher, Fairlawn, NJ), 2.5x SSC, 1% Roche blocking buffer (Roche, Indianapolis, IN), 0.1% yeast tRNA, (Invitrogen, Carlsbad, CA). Prehybridization and hybridizations were performed at 56^o^C using digoxygenin (DIG) labeled riboprobes (200ng/mL for culture, 400ng/mL for slice). Cells were washed for 1hr at 65^o^C in 2x SSC followed by 0.1x SSC at 65^o^C. Anti-DIG (sheep Fab fragments, Roche, Indianapolis, IN), anti-MAP2 and/or anti-NNAT antibodies were added and incubated for 1h at room temperature. For culture, tyramide signal amplification was performed for 5 min using FITC-TSA kit (Perkin-Elmer, Waltham, MA) followed by co-incubation with Cy3 goat anti-rabbit and Cy5 goat anti-mouse secondary antibodies [2]. For slice, Bio-Rad AP Substrate Kit (Hercules, CA) was used as previously described [3,4]. Slides were mounted using Fluoromount-G (Southern Biotech, Birmingham, AL).

***Immunofluorescent staining***

12-14*div* neurons were stained according to protocol used by the Martin lab at UCLA and described previously [2]. Dendrites were straightened and fluorescence intensity quantified using the Straighten plugin with ImageJ. Cells were treated as indicated with 2µM tetrodotoxin and 40µM APV in the presence or absence of 8µM actinomycin D, or 20µM anisomycin. Drugs were purchased from Ascent Scientific, Princeton, NJ. For slice sections, hippocampi from P21 brains were fixed and embedded in paraffin, cut into 5µm sections. Antigen retrieval was performed by boiling in 10mM sodium citrate, 0.05% Tween-20, pH 6 for 10m. Remaining steps were performed as described for cultured neurons. HeLa cells were counterstained with a three minute incubation in Propidium Iodide Solution (Sigma, St Louis MO).

***Western blotting***

Westerns were performed using antibody concentrations listed in Supplemental Information. Tissue was Dounce homogenized in the presence of a protease inhibitor cocktail (Sigma, St. Louis, MO) and/or Halt Phosphatase Inhibitor as indicated (Pierce, Rockford, IL). ECL Plus signal was detected using a Storm scanner (GE, Piscataway, NJ). Radioimmunoprecipitation assay (RIPA) buffer was used unless otherwise indicated.

***Calcium imaging***

Neurons grown on coverslips were transfected with GFP/NNATβ at 12*div* for 8h using Lipofectamine 2000 (Invitrogen, Carlsbad, CA) in growth media. Only one-quarter the recommended amount of Lipofectamine 2000 was used (e.g., for a 24-well plate, 0.5µL instead of 2µL was used). Following transfection, growth media was gently replaced with HEPES buffered solution (HBS) containing 129mM NaCl, 4mM KCl, 1mM MgCl_2_, 2mM CaCl_2_, 4.2mM glucose, and 10mM HEPES in the presence of 2µM TTX. pH was adjusted to 7.4 with NaOH, and osmolarity to 320 mOsm with sucrose as described by Korkotian and Segal [5]. Neurons were equilibrated in HBS for 30m at 26^o^C. HBS was then replaced by HBS containing 2µM Calcium Crimson-AM (Invitrogen, Carlsbad, CA). Cells were loaded with HBS+Calcium Crimson-AM at room temperature for 30 minutes, followed by several gentle washes of HEPES buffer to remove free dye. Images were taken similarly to Korkotian and Segal [6]. Transfected neurons were quickly identified and focused upon using GFP expression, then both Calcium Crimson and GFP scans were taken at 0.8ms/line using minimal laser intensity and 0.5µM optical sections (usually 2-4 sections per image). Due to a combination of short GFP overexpression and minimal laser intensity, we were unable to consistently image dendritic spines. Thapsigargin (Ascent Scientific, Princeton, New Jersey) was used at a final concentration of 1µM for 20 min. In experiments testing for additional glutamate-mediated calcium influx, cells were treated with 40µM glutamate for 5 min. For quantification, dendritic segments were straightened using the Straighten plugin in ImageJ and total fluorescent intensities quantified. Since Calcium Crimson-AM is not ratiometric, fluorescent signal was normalized to GFP fluorescence to control for bleaching and the level of NNATβ overexpression. Uniform Calcium Crimson loading was assessed *post hoc* by immunofluorescence using an antibody against BAPTA (Sigma, St. Louis, MO) as described previously (data not shown) [7].

***Synaptoneurosomes***

Synaptoneurosomes were prepared from 21*div* rat hippocampi as described in Muddashetty et al., 2007. SNS rested for 15m at 37^o^C, then were treated with anisomycin (40µM) for 15m when needed. SNS were then treated with 5µM NH125 (Sigma, St. Louis MO) or 2nM okadaic acid (Ascent Scientific, Princeton, NJ) for 30m at 37^o^C. 6x SDS protein loading buffer was then added to each sample followed by immediate heating of samples at 72^o^C for 5m.

***Semi-quantitative RT-PCR***

RNA was quantified by Nanodrop (Thermo Scientific, Wilmington, DE) and equal amounts reverse transcribed using Superscript III (Invitrogen, Carlsbad, CA) primed with random hexamers, followed by PCR with Phusion polymerase (New England Biolabs, Ipswich, MA). Samples were removed at 20, 25, 30 and 35 cycles. Images were taken using a Bio-Rad GelDoc (Hercules, CA) and quantified using ImageJ.

**References**

1. Banker G, Goslin K (1998) Culturing nerve cells. Cambridge, Mass.: MIT Press. xii, 666 p., 611 p. of plates p.

2. Poon MM, Choi SH, Jamieson CA, Geschwind DH, Martin KC (2006) Identification of process-localized mRNAs from cultured rodent hippocampal neurons. J Neurosci 26: 13390-13399.

3. Guzowski JF, Worley PF (2001) Cellular compartment analysis of temporal activity by fluorescence in situ hybridization (catFISH). Curr Protoc Neurosci Chapter 1: Unit 1 8.

4. Braissant O, Wahli W (1998) Differential expression of peroxisome proliferator-activated receptor-alpha, -beta, and -gamma during rat embryonic development. Endocrinology 139: 2748-2754.

5. Korkotian E, Segal M (2001) Spike-associated fast contraction of dendritic spines in cultured hippocampal neurons. Neuron 30: 751-758.

6. Korkotian E, Segal M (1998) Fast confocal imaging of calcium released from stores in dendritic spines. Eur J Neurosci 10: 2076-2084.

7. Tymianski M, Bernstein GM, Abdel-Hamid KM, Sattler R, Velumian A, et al. (1997) A novel use for a carbodiimide compound for the fixation of fluorescent and non-fluorescent calcium indicators in situ following physiological experiments. Cell Calcium 21: 175-183.
